# BACE1 inhibitory activity of fungal endophytic extracts from Malaysian medicinal plants

**DOI:** 10.1186/1472-6882-11-79

**Published:** 2011-09-24

**Authors:** Azzeme Harun, Richard Muhammad Johari James, Siong Meng Lim, Abu Bakar Abdul Majeed, Anthony LJ Cole, Kalavathy Ramasamy

**Affiliations:** 1 Collaborative Drug Discovery Research (CDDR) Group, Faculty of Pharmacy, Universiti Teknologi MARA (UiTM) Puncak Alam Campus, 42300 Bandar Puncak Alam, Selangor Darul Ehsan, Malaysia; 2 Brain Research Laboratory, Faculty of Pharmacy, Universiti Teknologi MARA (UiTM) Puncak Alam Campus, 42300 Bandar Puncak Alam, Selangor Darul Ehsan, Malaysia; 3 School of Biological Sciences, University of Canterbury, Private Bag 4800, Christchurch, New Zealand

## Abstract

**Background:**

BACE1 was found to be the major β-secretase in neurons and its appearance and activity were found to be elevated in the brains of AD patients. Fungal endophytic extracts for BACE1 inhibitory activity and cytotoxicity against PC-12 (a rat pheochromocytoma with neuronal properties) and WRL68 (a non-tumorigenic human hepatic) were investigated.

**Methods:**

Endophytes were isolated from plants collected from Kuala Pilah, Negeri Sembilan and the National Park, Pahang and the extracts were tested for BACE1 inhibition. For investigation of biological activity, the pure endophytic cultures were cultivated for 14 days on PDA plates at 28°C and underwent semipolar extraction with ethyl acetate.

**Results:**

Of 212 endophytic extracts (1000 μg/ml), 29 exhibited more than 90% inhibition of BACE1 in the preliminary screening. Four extracts from isolates HAB16R13, HAB16R14, HAB16R18 and HAB8R24 identified as *Cytospora rhizophorae *were the most active with IC_50(BACE1) _values of less than 3.0 μg/ml. The most active extract HAB16R13 was shown to non-competitively inhibit BACE1 with *K*_i _value of 10.0 μg/ml. HAB16R13 was considered non-potent against PC-12 and WRL68 (IC_50(CT) _of 60.0 and 40.0 μg/ml, respectively).

**Conclusions:**

This first report on endophytic fungal extract with good BACE1 inhibitory activity demonstrates that more extensive study is required to uncover the potential of endophytes.

## Background

Alzheimer's disease (AD) is the most common cause of dementia in elderly people, and the fourth most common cause of death in developed countries [1]. It is estimated that about 18 million people worldwide are currently affected by this disease and this figure is projected to double by 2025 with an ageing population [2].

Patients diagnosed with AD suffer memory loss, language deterioration, poor judgment and impaired visuospatial capability [3]. At present, there is no cure for AD. Medication for AD only helps slow down progression of the disease so as to improve patients' quality of life. Histopathologically, AD is characterized by the formation of neurofibrillary tangles (NFT) from phosphorylated tau protein in the neurons and the deposition of β-amyloid (Aβ) plaque in the parenchyma of the amygdale, hippocampus and neocortex of the brain [4]. The major component of amyloid plaque is the β-amyloid protein (Aβ), a 39-43 amino acid peptide composed of a portion of the transmembrane domain and the extracellular domain of the amyloid precursor protein (APP) [5]. Aβ is produced by a sequential cleavage of APP at the amino terminal end by β-secretase followed by γ-secretase at the carboxyl terminal end [6]. β-secretase has been identified as an aspartic protease, β-site amyloid precursor protein cleaving enzyme 1 (BACE1), also called Asp 2 (for novel aspartic protease 2) and memapsin 2 (for membrane aspartic protease/pepsin 2). It is currently the most attractive target for the inhibition of amyloid production since it is the key enzyme that initiates the formation of Aβ [7]. Furthermore, BACE1 was found to be the major β-secretase in neurons [8] and its expression and activity were found to be elevated in the brains of AD patients.

β-secretase inhibitors have potential to be developed as anti-dementia drugs. Nevertheless, all drugs considered for AD must be able to cross the plasma membrane, and most importantly the blood-brain-barrier [9]. Enzyme inhibitors with therapeutic potential should preferably be smaller than 700 Da, making large peptide-based inhibitors not viable as drug candidates [10]. Thus, the secondary metabolites of plants and microbes, which have relatively low-molecular weights and high lipophilicity, may offer possibilities for drugs against AD [10].

Plants serve as a reservoir of microorganisms known as endophytes [11]. These endophytes, mostly fungi and bacteria, live in the intercellular spaces of plant tissues. Endophytes have high diversity and are relatively fast growing on routinely used laboratory media. Many of the endophytes species are able to produce large amount of novel compounds and is predicted to be a major source for new and useful metabolites [12]. To the best of our knowledge, their ability to produce compounds that inhibit β-secretase have not been previously reported.

## Methods

### Fungi

Fungal endophytes were obtained from the culture collection of the Collaborative Drug Discovery Research (CDDR) Group, Faculty of Pharmacy, Universiti Teknologi MARA (UiTM), Malaysia. They were previously isolated from medicinal plants from rainforest parks in Malaysia (Kuala Pilah, Negeri Sembilan [13], and the National Park, Pahang [14]). Axenic cultures were maintained on potato dextrose agar (PDA, Oxoid) plates. Extracts of cultures grown for 14 days at 28°C on PDA plates were assessed for bioactivity. A total of 212 strains were grown for investigation.

### Semipolar extraction of fungal cultures

Semipolar extraction was as described previously by Ramasamy *et al. *[13]. Briefly, after 14 days of incubation, 10 plates of each isolate were transferred into a conical flask (500 ml) and homogenized using a homogenizer (Kika Labortechnik, T25). Ethyl acetate (200 ml) was added and left to stir overnight at room temperature. The mixture was filtered through Whatman No.1 filter paper, after which sodium sulphate was added to remove the aqueous layer within the mixture. The mixture was then transferred to a round bottom flask (500 ml) and dried using a rotary evaporator (Buchi, R-215). The resultant extract was dissolved in 1 ml dimethyl-sulfoxide (DMSO) (Sigma) and stored at -20°C until use.

### BACE1 inhibitory activity

Crude endophytic extracts (1000 μg/ml) were assayed for BACE1 inhibition using a fluorescence resonance energy transfer (FRET) assay (Pan Vera Co.), that uses baculovirus-expressed BACE-1 and a specific substrate (Rh-EVNLDAEFK-quencher) based on the Swedish mutation of the amyloid precursor protein (APP). This peptidic substrate becomes highly fluorescent upon enzymatic cleavage. A mixture of 10 μl of BACE1 substrate (Rh-EVNLDAEFK quencher, in 50 nmol/l ammonium bicarbonate), 10 μl of test compound, and 10 μl of BACE1 (β-secretase) enzyme [(50 mM Tris (pH7.5), 10% glycerol) (1.0 U/ml)] were incubated for 60 min at room temperature and protected from light. Then 10 μl of BACE1 stop buffer (2.5 mol/l sodium acetate) was added to the mixture. Fluorescence was read using a multiwell spectrofluorometer (infiniteM200, TECAN) under excitation at 545 nm and the emitted light at 585 nm. Percentage inhibition of the enzyme was then calculated. In the preliminary screening all 212 endophytic extracts were tested for BACE1 inhibition using a single concentration of 1000 μg/ml. Active extracts showing >90% BACE1 inhibition in preliminary screening were then reexamined at a concentration ranging from 0.1 μg/ml to 1000 μg/ml to determine the IC_50(BACE1) _values_. _The IC_50 (BACE1) _value is defined as the concentration of BACE1 inhibitor that is required to inhibit 50% of BACE1 activity.

### Determination of the inhibition pattern on BACE1

To investigate the BACE1 inhibition pattern, the most active endophytic extract (HAB16R13) was added to each reaction mixture. The BACE1 inhibitory activity was measured at different concentrations of substrate. The inhibition constant (*K*_i_) of β-secretase inhibitor was then calculated using Dixon plots. The reaction velocity is measured at a fixed concentration of substrate but with different extract concentrations (from 0.1 μg/ml to 1000 μg/ml). The 1/V0 (RFU/min) against inhibitor concentration was plotted and the kinetics of BACE1 in the presence of the inhibitor determined.

### Cytotoxicity

PC-12 (ATCC CRL-1721), a rat pheochromocytoma was maintained in DMEM high glucose (PAA Laboratories) supplemented with 10% horse serum, 5% heat-deactivated foetal bovine serum (FBS) and 1% penicillin/streptomycin. PC-12 has neuronal properties and expression of BACE1 is readily detectable in neuronal cells [15-17]. WRL68 (ATCC CL-48), a non-tumorigenic fetal hepatic cell was maintained in RPMI 1640 (Sigma) supplemented with 10% heat-deactivated foetal bovine serum (FBS) (PAA Laboratories) and 1% penicillin/streptomycin (PAA Laboratories). Cells of about 80-85% confluence were harvested and plated onto 96-flat bottom well plates for experimental use. The culture was maintained in a humidified incubator at 37°C in an atmosphere of 5% CO_2._

The extract showing the greatest BACE1 inhibitory activity HAB16R13 was then tested (at various concentrations from 0.1 μg/ml - 1000 μg/ml) for cytotoxicity against PC-12 and WRL68 using the MTT assay [18]. Plates were read using an Elisa plate reader (Perkin Elmer) at 520 nm. Data generated was used to plot a dose-response curve of which the concentration of extract required to kill 50% of the cell population (IC_50 (CT)_) was determined.

### DNA extraction, PCR amplication and sequencing

The active isolates (HAB16R13, HAB16R14, HAB16R18 and HAB8R24) were identified. using molecular methods. Fungal DNA extraction was carried out using MasterPure Yeast DNA Purification Kit (Epicentre, USA). 18S ribosome DNA sequence was isolated by PCR using Internal Transcribed Spacer ITS-1 primer (TCCGTAGGTGAACCTGCGG) and ITS-4 (5'-TCCTCCGCTTATTGATATGC-3'). The amplification was performed in a total reaction volume of 100 μl containing 50 μl of GoTaq Green Master Mix (Promega, USA), 4 μl of each of the primer, 6 μl of DNA template and 36 μl of nuclease free water using Applied Biosystems GeneAmp PCR System 9600 (Biorad, USA). PCR condition consisted of an initial denaturing step of 1 min at 94°C followed by 40 cycles of 95°C for 30 s, annealing at 50°C for 1 min and 72°C for 30 s. The reaction was completed with final extension at 72°C for 7 min and at 25°C for 30 s. The amplified PCR product was separated by electrophoresis in 1.5% (w/v) agarose gel at 90 V for 40 min in 1× Tris-acetate-EDTA (TAE) buffer, stained with ethidium bromide and visualized under UV light and photographed. A 100-bp size marker (Promega, USA) was used as reference. The amplified PCR product was purified using Wizard^® ^SV Gel and PCR Clean-Up System (Promega, USA) and subsequently sent for sequencing. The analysis and comparison of the sequences were performed with nucleotide blast of GenBank (http://www.ncbi.nlm.nih.gov). The sequences were deposited in GenBank.

### Phylogenetic analysis

The ITS sequences were aligned to each other as well as the sequences retrieved from the NCBI databases, using multiple sequence alignment software CLUSTAL W program with default settings [19]. Phylogenetic analyses were performed by the neighbour-joining (NJ) method using Molecular Evolutionary Genetic Analysis 4 (MEGA4) software [20]. Parsimony trees were obtained using the Close-Neighbor-Interchange algorithm with search level 3, in which the initial trees were obtained with ten random addition replicates of the sequences [21,22]. All positions containing gaps and missing data were eliminated from the dataset (complete deletion option). Tree stability was evaluated by 1000 parsimony bootstrap replicates [21]. Branches corresponding to partitions reproduced in less than 50% bootstrap replicates were collapsed. A phylogenetic tree was constructed from distance matrix values by the neighbour-joining (NJ) method using the p-distance parameter model to estimate evolutionary distance. A bootstrap analysis was performed using 1000 resamples of the data. *Phomopsis theae *isolate NW284w was used as an outgroup.

### Statistical analysis

Differences between the extracts were evaluated using the one-way ANOVA procedure in SPSS version 16.0. When there was a difference, the LSD (least significant different) post hoc test was used to identify pairs that differed significantly. Significance was *P < 0.05 *unless otherwise stated.

## Results

### BACE1 inhibitory activity

BACE1 inhibitory activity of 212 endophytic extracts (1000 μg/ml) in preliminary screening found 13.7% (29) to be very active with > 90% inhibition of BACE1 activity. A further 13.7% (29) of the extracts also showed very good activity (80-89.9% inhibition). 14.2% (30) and 32.5% (69) of the extracts displayed 70-79.9% and 50-69.9% inhibition, respectively. The remaining 25.9% (55) exhibited <50% inhibition of BACE1 activity. IC_50 (BACE1) _values of 29 of the most active strains are shown in Table 1. Four extracts, HAB16R13, HAB16R18, HAB16R14 and HAB8R24 exhibited IC_50 (BACE1) _values of less than 3.0 μg/ml. HAB16R13 (IC_50 (BACE1) _= 2.15 μg/ml), showed the best BACE1 inhibitory activity.

**Table 1:** BACE1 inhibitory activity (IC_50 (BACE1)_) of active endophytic extracts

Strain	**IC**_**50(BACE1) **_**(μg/ml) ± SD**
HAB16R13	2.15 ± 0.49^a^
HAB16R18	2.40 ± 0.14^b^
HAB16R14	2.85 ± 0.91^b^
HAB8R24	2.85 ± 0.07^b^
HAB16R12	3.10 ± 0.84^b^
HAB6S14	5.25 ± 0.35^b^
HAB15R7	6.00 ± 2.12^b^
HAB16R15	6.15 ± 2.01^b^
KK9R1	6.20 ± 1.69^b^
HAB16R11	6.45 ± 0.77^b^
HAB6S11	7.00 ± 4.24^b^
HAB13S18	7.75 ± 0.35^b^
HAB4L5	8.50 ± 3.53^b^
HAB6R8	9.25 ± 1.06^b^
HAB4L3	11.00 ± 4.42^b^
KT36L1	11.10 ± 1.27^b^
HAB15R6	11.50 ± 2.12^b^
HAB16L32	11.50 ± 0.70^b^
KK11S3	12.50 ± 3.53^b^
KT39R1	16.50 ± 4.94^b^
HAB26S6	19.00 ± 1.41^b^
KT44S3	25.00 ± 7.07^b^
KT34L2	34.00 ± 7.07^b^
HAB8R19	40.00 ± 14.14^c^
HAB13L4	42.50 ± 3.53^c^
HAB13L2	45.00 ± 7.07^c^
HAB12S12	55.00 ± 7.07^c^
HAB13S13	57.50 ± 24.74^c^
HAB13R29	80.00 ± 28.28^c^

^a-c ^Means with no common superscript differ significantly (P < 0.05).

### Determination of the inhibition pattern on BACE1

**Figure 1:**
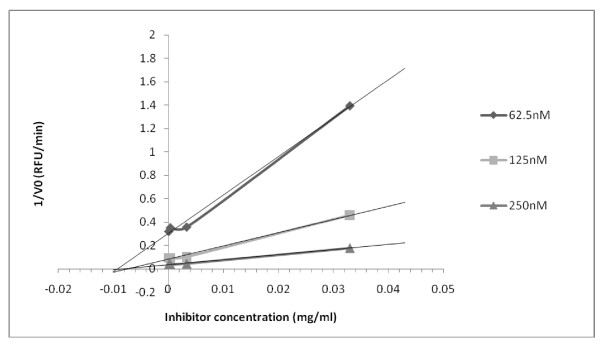
**Dixon plot to determine the inhibition constants of HAB16R13 against BACE1 at various substrate concentrations**.

The inhibition pattern displayed by the most active HAB16R13 endophytic extract was then studied. Plots of the initial velocity versus endophytic extract concentrations in the presence of different substrate concentrations gave a family of straight lines. The inhibition pattern of extract HAB16R13 (*K*_i _= 10 μg/ml) against BACE1 from the Dixon plot (Figure 1) was found to be non-competitive with the substrate at the active site of BACE1. It may bind to either another regulatory site or to the subsite of β-secretase.

### Cytotoxicity

**Figure 2:**
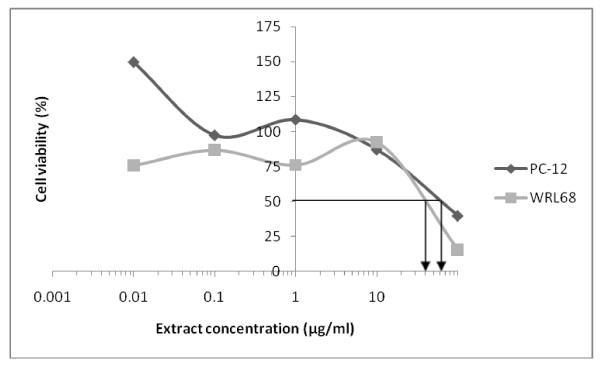
**Cytotoxic effects of HAB16R13 against PC-12 and WRL68 cell lines**. Each point is expressed as the mean of three independent experiments.

Extract HAB16R13 when tested against PC-12 and WRL68 showed IC_50 (CT) _values of 60.0 and 40.0 μg/ml respectively (Figure 2), which are considered to be non-potent. The criterion established by the US NCI is that crude extract with IC_50 (CT) _value of less than 20 μg/ml is considered to have *in vitro *cytotoxicity [23].

### Molecular identification and phylogenetic analysis

**Figure 3:**
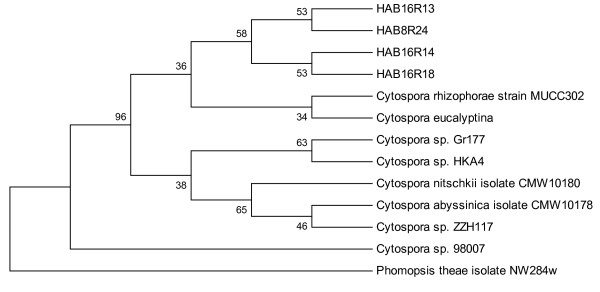
**Tree obtained by heuristic search in phylogenetic analysis of the ITS regions of the rDNA of, HAB16R13, HAB16R14, HAB16R18 and HAB8R24 as well as reference isolates**. *Phomopsis theae *isolate NW284w is used as outgroup. Bootstrap confidence level based on 1000 resamples is given on the appropriate branches.

The ITS of HAB16R13, HAB16R14. HAB16R18 and HAB8R24 were found to be 586-593 bp in length. A BLAST search of the ITS of all four isolates revealed that they were nearly identical to *Cytospora rhizophorae *(98-99% similarity). A further phylogenetic analysis based on ITS sequences was conducted to compare the sequences with those in GenBank to determine their relationship and authenticate the identification. There was a total of 481 positions in the final dataset, out of which 37 were parsimony informative (Figure 3). Similar results were obtained using neighbour-joining analyses (results not shown). Most of the clades were supported by bootstraps values (34-96% bootstrap support). All four isolates were found to be in the same clade with *Cytospora rhizophorae *strain MUCC302 and *Cytospora eucalyptina*. *Cytospora rhizophorae *is from the class Ascomycetes, order Diaporthales and family Valsaceae.

## Discussion

Endophytic fungi, HAB16R13, HAB16R14 and HAB16R18 were isolated from the roots of *Cinnamomum porrectum *while HAB8R24 from *Polyalthia glauca*. Oil from the root of *C. porrectum *has been documented to exhibit antimicrobial activity [24] and *Polyalthia *sp. used as an aphrodisiac, anti-parasite, anti-rheumatic and as an anti-inflammatory agent [25]. Although the compounds responsible for the BACE1 inhibitory activity were not identified in the present study, *Cytospora *sp. has been reported to produce cytosporacin [26], grahamimycin A [27], cytoskyrin A [28] and cytosporone E [29]. These compounds have been reported to exhibit antimicrobial activity [27-30]. Interestingly, cytosporic acid was found to inhibit a critical enzyme involved in the replication of HIV with an IC_50 _of 20 μM [28]. The pure compound cytoskyrin A, displayed poor cytotoxicity against some tumor cell lines (IC_50_> 5 μg/ml) *in vitro *[29]. Grahamimycin A also did not induce any toxic symptoms in adult mice [27].

Many groups have focused on high-throughput screening of chemical libraries for BACE1 inhibitors but discovery of naturally occurring BACE1 inhibitors have been limited. Thus far only one BACE1 inhibitor drug candidate (CTS-21166) has completed the Phase 1 clinical trial [31]. Several hydroxyl-containing compounds (hydroxyethylene and hydroxyethylamine) have been reported as BACE1 inhibitors [32,33]. Chitosan derivatives from crab shell and latifolin from *Dalbergia sissoo *have shown weak BACE1 inhibition [34,35]. Catechins from green tea [13], ellagic acid and punicalagin from pomegranate [36], hispidin from mycelial cultures of *Phellinus linteus *[37] and several compounds isolated from *Sanguisorbae radix *[38] have all been studied as BACE1 inhibitors. In order to have therapeutic potential, however efficacious penetration of the blood-brain barrier requires the molecular weights of inhibitors to be under 700 Da making large peptide-based inhibitors unsuitable as drug candidates. Thus, plants and microbial metabolites that have relatively low molecular weights and lipophilicity may be suitable as drug candidates for BACE1 inhibition [39].

Based on the prevailing view on the fundamental role of Aβ in the pathogenesis of AD, there seems to be good reason to expect that β-secretase inhibitor drugs may alter the course of the disease [31]. This first report of a fungal endophyte producing a BACE1 inhibitor would indicate they warrant further investigation.

## Conclusion

In conclusion, this preliminary screening of fungal endophytic extracts revealed their potential as a source of BACE1 inhibitors which could have a role in the development of drugs for treatment of neurodegenerative diseases.

## Authors' contributions

KR was the principal investigator who participated in the designing of the study and writing of the manuscript. AH participated in overall conduction of experiments and writing the manuscript. RMJJ, LSM and ALJC participated in the planning of the study and writing the manuscript. ABAM participated in the planning of the study. All authors have read and approve the final manuscript.

## Competing interests

The authors declare that they have no competing interests.

## Pre-publication history

The pre-publication history for this paper can be accessed here:

http://www.biomedcentral.com/1472-6882/11/79/prepub
